# An Overview of *Hox* Genes in Lophotrochozoa: Evolution and Functionality

**DOI:** 10.3390/jdb4010012

**Published:** 2016-03-19

**Authors:** Marco Barucca, Adriana Canapa, Maria Assunta Biscotti

**Affiliations:** Dipartimento di Scienze della Vita e dell’Ambiente, Università Politecnica delle Marche, via Brecce Bianche, 60131 Ancona, Italy; a.canapa@univpm.it (A.C.); m.a.biscotti@univpm.it (M.A.B.)

**Keywords:** *Hox*, Lophotrochozoa, Mollusca, Annelida, Nemertea, Platyhelminthes

## Abstract

*Hox* genes are regulators of animal embryonic development. Changes in the number and sequence of *Hox* genes as well as in their expression patterns have been related to the evolution of the body plan. Lophotrochozoa is a clade of Protostomia characterized by several phyla which show a wide morphological diversity. Despite that the works summarized in this review emphasize the fragmentary nature of the data available regarding the presence and expression of *Hox* genes, they also offer interesting insight into the evolution of the *Hox* cluster and the role played by *Hox* genes in several phyla. However, the number of genes involved in the cluster of the lophotrochozoan ancestor is still a question of debate. The data presented here suggest that at least nine genes were present while two other genes, *Lox4* and *Post-2*, may either have been present in the ancestor or may have arisen as a result of duplication in the Brachiopoda-Mollusca-Annelida lineage. Spatial and temporal collinearity is a feature of *Hox* gene expression which was probably present in the ancestor of deuterostomes and protostomes. However, in Lophotrochozoa, it has been detected in only a few species belonging to Annelida and Mollusca.

## 1. Introduction

*Hox* genes, a subfamily of homeobox genes, encode transcription factors containing a highly conserved 60 amino acid homeodomain characterized by a helix-turn-helix motif [1]. The members of this gene subfamily are regulators of animal embryonic development and play a role in the patterning of the anterior-posterior body axis of Bilateria [2].

*Hox* genes were first discovered in *Drosophila melanogaster* where they are organized in the split *Antennapedia-Bithorax* complex located on chromosome 3 [3,4]. In some cases, these genes are arranged in clusters, and, hence, they are physically linked on the same chromosome. The number of clusters varies in agreement with the genome duplications that the organisms experienced during evolution, ranging from one in protostomes and invertebrate deuterostomes to four in sarcopterygians which experienced two rounds of whole genome duplication (WGD), with as many as seven in teleosts which experienced a third lineage specific event of WGD (Teleost Specific Genome Duplication, TSGD) [5].

Generally, one feature of the *Hox* cluster is spatial collinearity: the gene order on chromosomes reflects the order of gene expression and function. The genes at the 3′ end of the cluster are expressed in the anterior part of the body while those at the 5′ end in the posterior part. Collinearity may also be temporal, meaning that the genes at the 3′ end are expressed before those at the 5′ end [6]. This feature is more evident in bilaterian organisms displaying an unbroken cluster than in others which show dispersed or broken clusters [7,8,9,10].

On the basis of gene position and gene function, the *Hox* cluster can be subdivided into four classes [11]: anterior class, Paralog Group 3, central class, and posterior class. The composition of these classes vary across taxa due to duplication, inversion, or gene loss events that occurred during evolution [10,12,13,14]. Moreover, fragmented clusters may also be related to the presence of transposable elements that could promote chromosomal rearrangements [15].

Although *Hox* genes show a high sequence similarity, they play a remarkable role in the wide morphological diversity of animals [10]. One of the major groups of bilaterian organisms is Protostomia, which is subdivided into two clades: Ecdysozoa and Lophotrochozoa. The peculiarity of the former is the ability to undergo ecdysis under the hormonal control of ecdysteroids. The latter clade is characterized by the trochophore, the free-swimming ciliated larvae, and/or the lophophore, the feeding structure made up of tentacles surrounding the mouth of adults.

Regarding *Hox* cluster composition, besides the *Hox* genes belonging to the *Paralog Group-1* (*PG-1*), *PG-2*, *PG-3*, *PG-4,* and *PG-5*, Ecdysozoa also exhibit *ftz*, *Antp*, *Ubx*, *abd-A*, and *Abd-B*, while Lophotrochozoa also include *Lox5*, *Lox2*, *Lox4*, *Post-1* and *Post-2* [16,17].

This paper focuses on Lophotrochozoa which are characterized by a high diversity of body architecture, and are, therefore, ideal for studying the evolution of development. This review presents an overview of the presence and expression patterns of *Hox* genes in 12 lophotrochozoan phyla. The data obtained allowed different hypotheses to be delineated regarding the evolution of the *Hox* gene subfamily within Lophotrochozoa and its implications on development.

## 2. Hox Presence in Lophotrochozoa

Body plan evolution and diversification in metazoans have not only been related to changes in *Hox* cluster composition such as cluster and gene duplications, and gene loss, but also to gene expression and regulatory interactions [14]. An understanding of *Hox* gene cluster composition can provide insight into the evolutionary history that these genes have undergone within Lophotrochozoa.

**Figure 1:**
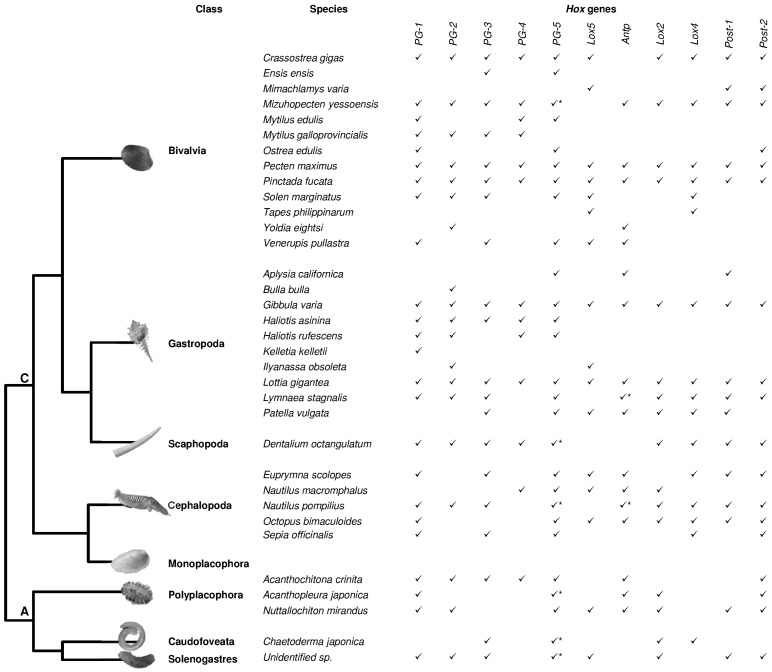
*Hox* genes in Mollusca. *Hox* genes identified in the Mollusca phylum are reported. Tree topology following Smith *et al.* [92,93]. C: Conchifera; A: Aculifera. * indicates duplicated genes probably due to erroneous attribution [94]. The graphics of the figure were modified from Biscotti *et al.* [94]. For references see Table S1.

**Figure 2:**
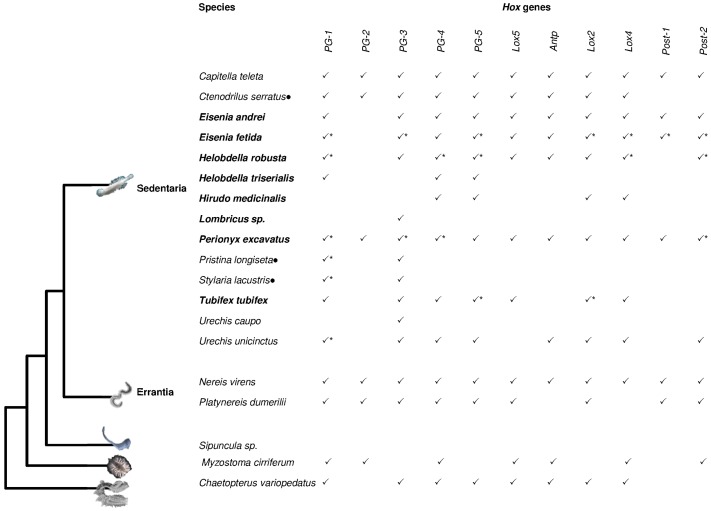
*Hox* genes in Annelida. *Hox* genes identified in the Annelida phylum are reported. Tree topology following Struck *et al.* [95]. * indicates duplicated genes. Black dots indicate species not included in the analysis carried out by Struck *et al.* [95]. Species in bold belong to Clitellata. For references see Table S2.

**Figure 3:**
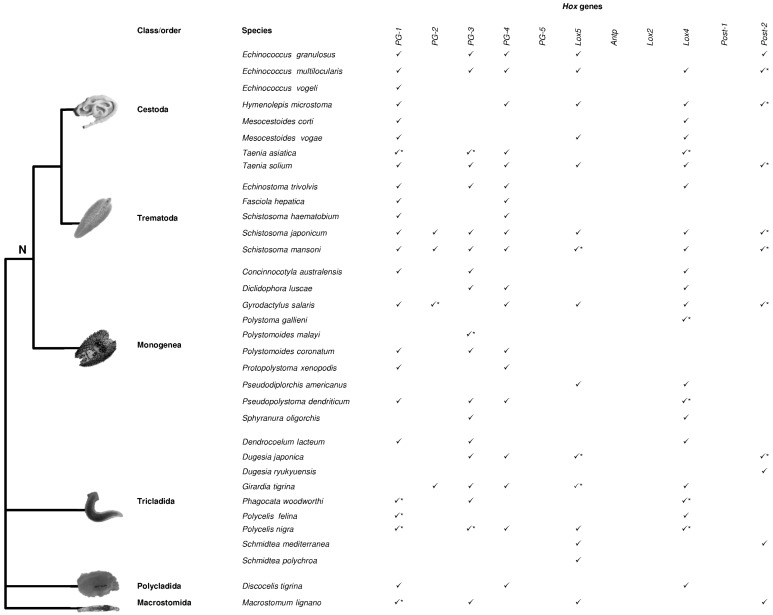
*Hox* genes in Platyhelminthes. *Hox* genes identified in the Platyhelminthes phylum are reported. The tree is modified from Hahn *et al.* [32]. N: Neodermata. * indicates duplicated genes. The duplication reported for *PG-2* gene in *Gyrodactylus salaris* might be due to an erroneous attribution of *PG-3* gene. For references see Table S3.

Although internal relationships within the Lophotrochozoa clade are still controversial [18,19,20,21,22], we focused on works concerning the presence of *Hox* genes in 12 phyla, and the evolution of the genes composing the *Hox* cluster was discussed in relation to the evolutionary relationships between lophotrochozoan phyla. The works summarized here clearly indicate that data on *Hox* genes are rather scarce for Lophotrochozoa, and a limited number of works on complete genomes have provided insight into *Hox* gene cluster composition [20,22,23,24,25,26,27,28,29,30,31,32,33]. About half of the lophotrochozoan phyla have never been investigated while only one species has been analyzed in Brachiopoda and Bryozoa, two species in Rotifera, and three species in Nemertea (Table 1) [16,22,23,34,35,36,37]. More information is available for the three major lophotrochozoan phyla: Mollusca (about 30 species, Figure 1, Table S1) [16,20,24,26,38,39,40,41,42,43,44,45,46,47,48,49,50,51,52,53,54,55,56,57,58], Annelida (about 20 species, Figure 2, Table S2) [8,16,20,46,59,60,61,62,63,64,65,66,67,68,69,70,71,72,73] and Platyhelminthes (about 30 species, Figure 3, Table S3) [27,28,29,30,31,32,33,74,75,76,77,78,79,80,81,82,83,84,85,86,87,88,89,90].

The Mollusca phylum is divided into eight classes: Solenogastres, Caudofoveata, and Polyplacophora belonging to Aculifera and Bivalvia, Cephalopoda, Gastropoda, Monoplacophora, and Scaphopoda belonging to the sister taxa Conchifera [92,93]. Mollusks include organisms living in different ecological niches from marine to freshwater and terrestrial environments. Their morphology is extremely variable, ranging from Aplacophora with a wormlike appearance and no shell to Cephalopoda which have a well-developed cephalic region and have co-opted the mantle for locomotion. The muscular foot also presents different morphologies as it is adapted for a variety of functions. With the exception of Monoplacophora, for which no data are available, *Hox* genes have been identified in a single species of Scaphopoda [40], two species of Aplacophora [40], three species of Polyplacophora [40,57,58], five species of Cephalopoda [26,40,46,55,56], 10 species of Gastropoda [16,20,40,46,47,48,49,50,51,52,53,54], and 13 species of Bivalvia [24,38,39,40,41,42,43,44,45] (Figure 1, Table S1). Our previous review [94] provided evidence that all 11 genes of the *Hox* lophotrochozoan cluster are found within Bivalvia, Cephalopoda, and Gastropoda, which are the three most studied molluskan classes. Moreover, genome sequencing has revealed, for the first time in lophotrochozoans, that the gastropod *Lottia* *gigantea* presents an intact cluster [20]. In the genome assembly of the Pacific oyster, *Crassostrea gigas*, the *Antp* gene is clearly missing and *Hox* genes are located on four scaffolds [24,38]. In the pearl oyster *Pinctada fucata,* all 11 *Hox* genes are present and clustered on three scaffolds. Moreover, non-*Hox* genes are present in the *Hox* clusters of both the above-mentioned oysters, thereby suggesting that this is a feature which occurred in their common ancestor [25].

In the genome of the cephalopod, *Octopus bimaculoides* eight of the 11 *Hox* genes (with the exception of *Hox2*, *Hox3* and *PG-4*) have been identified and located on eight separate scaffolds [26].

Annelida is another large and morphologically diverse taxon of Lophotrochozoa. Traditionally, this phylum includes segmented worms but currently Myzostomida [95], Echiura, and Sipuncula are also considered as Annelida. The two latter taxa show segmentation only at the larval stage [96,97].

A recent phylogenomic analysis divides Annelida into two main groups reflecting different lifestyles, Errantia and Sedentaria, with Sipuncula and Myzostomida which occupy a basal position (Figure 2) [95].

Within the Sedentaria group, the species belonging to Clitellata exhibit a highly dynamic *Hox* gene cluster which is broken into several genomic regions and characterized by gene duplication and loss events. In fact, extensive studies on the leech *Helobdella* *robusta* [20] and two earthworms, *Eisenia fetida* [61] and *Perionyx excavatus* [67], suggest that individual duplications, large segmental duplications and/or whole genome duplications could have played a pivotal role in determining the current *Hox* gene number and arrangement in these lineages. Moreover, the ortholog *Post-1* gene has not been retrieved in leeches and the *PG-2* gene seems be absent in Clitellata with the exception of *P. excavates* [67]. These organisms exhibit a different number of segments, internal anatomy, and shapes. Therefore, it has been suggested that differences in presence, absence, and the arrangement of *Hox* genes may be responsible for the evolution of the annelid body plan diversity [20].

The genomic survey performed in *Capitella* *teleta* indicates that *Hox* genes are located on three scaffolds although duplication events have not been detected [8,20].

In general, the common ancestor of Sedentaria and Errantia had all 11 genes of the *Hox* cluster. The species *Myzostoma cirriferum* and *Chaetopterus variopedatus*, which are basal in the annelid phylogeny, do not show a complete cluster although whole genome sequencing has not yet been performed for these species.

**Figure 4:**
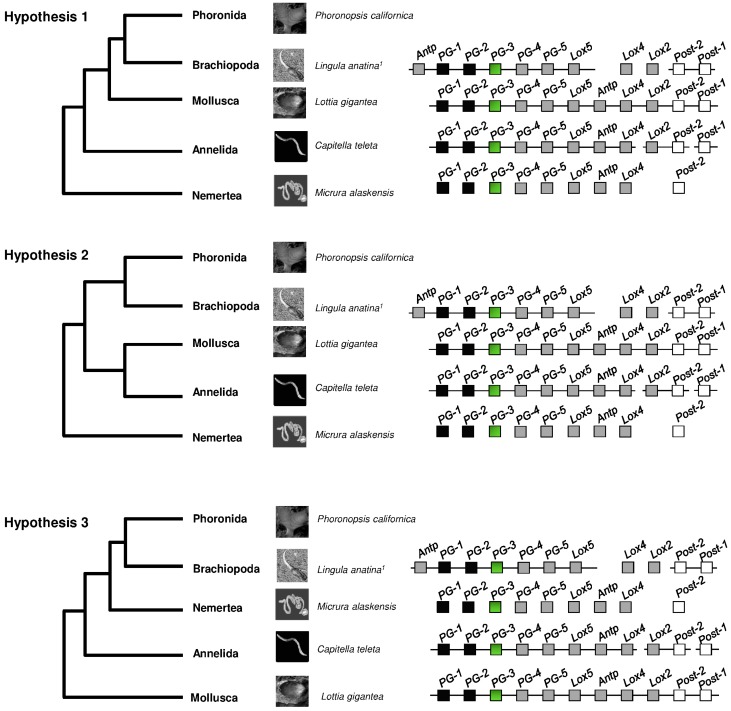
Hypotheses on phylogenetic relationships between the major lophotrochozoan phyla. In the trees displaying the three hypotheses [22], the species in which the largest numbers of *Hox* genes have been identified are reported for Nemertea and Brachiopoda, for Mollusca *Lottia* *gigantea*, the only species showing the intact cluster in Lophotrochozoa, for Annelida *Capitella* *teleta*, the species to date showing the least fragmented cluster. Lines underneath boxes indicate syntenic arrangement. Split lines indicate that the cluster is located on different chromosomes or scaffolds. The length of the depicted cluster is not proportional to the effective length in genomes. ^1^: in *Lingula anatina, Lox4* and *Lox2* genes have been identified in de Rosa *et al.* [16]. Dark boxes indicate genes belonging to the Anterior class; green boxes indicate genes belonging to the *Paralog Group 3*; grey boxes indicate genes belonging to the Central class; white boxes indicate genes belonging to the Posterior class. Gene abbreviations: *Antp*: *Antennapedia*; *lab*: *labial*; *Lox2: Lophotrochozoa Hox2*; *Lox4*: *Lophotrochozoa Hox4*; *Lox5*: *Lophotrochozoa Hox5*; *pb*: *Proboscipedia*; *PG-1: paralog group 1*; *PG-2*: *paralog group 2*; *PG-3*: *paralog group 3*; *PG-4: paralog group 4*; and *PG-5*: *paralog group 5*; *Post-1*: *Posterior-1*; *Post-2*: *Posterior-2*. The graphics of the figure were modified from Biscotti *et al.* [94]. For references see Table 1, Tables S1 and S2.

Within Lophotrochozoa, the phylogenetic relationships of Annelida and Mollusca with respect to Brachiopoda, Phoronida, and Nemertea are still a question of debate. Recently, three hypotheses have been proposed regarding the phylogenetic relationships between these taxa (Figure 4): according to the first hypothesis, Brachiozoa (Brachiopoda + Phoronida) is a sister group to Mollusca while Nemertea occupies a basal position in all the taxa considered [22,98]; the second hypothesis suggests that Brachiozoa is a sister group to Mollusca + Annelida [99]; the third hypothesis considers Annelida as a sister group to the clade including Brachiozoa and Nemertea [21].

*Lox2* and *Post-1* genes have not yet been recovered in three nemertean species, *Lineus sanguineus* [35], *Micrura alaskensis* [36], *Pantinonemertes californiensis* [37]. According to the evolutionary hypotheses 1 [22,98] and 2 [99], the duplication events leading to these two genes occurred after the separation of Nemertea and other lophotrochozoan phyla, thereby suggesting that the common ancestor of Nemertea and Annelida-Mollusca-Brachiopoda had nine genes [36,37,94]. Alternatively, considering evolutionary hypothesis 3, the absence of *Lox2* and *Post-1* might be due to a secondary loss.

The phylum Platyhelminthes is the fourth largest animal phylum after arthropods, mollusks and chordates. Members of the phylum Platyhelminthes have a simple bilateral body plan, characterized by the absence of traits found in most bilaterians (e.g., coelom, anus, circulatory and respiratory systems) and for this reason they were previously considered as basal bilaterians [100]. However, the presence in many flatworms of spiral embryonic cleavage, which is a mode of development present in other invertebrate phyla, such as annelids or mollusks, has made their classification very controversial. Platyhelminthes are currently considered as part of Lophotrochozoa, with the acoel and nemertodermatid flatworms separate from the Platyhelminthes and considered as the earliest branching extant bilaterians. However, some phylogenetic studies regard this taxon as separate from Lophotrochozoa [21,98]. The Platyhelminthes are divided into three classes, the free-living class “Turbellaria” and two parasitic classes, Cestoda and Trematoda. Subsequently, the parasitic species are subdivided into three classes, Trematoda, Cestoda, and Monogenea (together called Neodermata), while the class “Turbellaria”, a paraphyletic group, is divided into about 11 orders (depending on the authors) [100].

In Platyhelminthes, the *Hox* gene set is reduced (Figure 3, Table S3), with *PG-5*, *Antp*, *Lox2*, and *Post-1* being absent in all the considered taxa [27,28,29,31,32]. The lack of these genes even in free-living flatworms suggests that their absence may not be related to the parasitic lifestyle of many species of this phylum. In Cestoda, the *PG-2* gene seems to be absent and, among the other species examined, orthologs have only been reported in four species: *Girardia tigrina*, *Schistosoma mansoni*, *S. japonicum*, *Gyrodactylus salaris* (Figure 3). *PG-1*, *PG-3*, *Lox5*, *Lox4*, and *Post-2* genes show duplications in several species belonging to all groups. A duplication has been detected for *PG-2* in *G. salaris* although one of the two genes might belong to *PG-3*; indeed, in Platyhelminthes, *PG-2* and *PG-3* genes share several conserved amino acidic residues that might make correct gene attribution difficult [28,75].

Moreover, as regards *Hox* gene arrangement, genome surveys have shown that the cluster is disrupted. The developmental strategies of some of these organisms with a parasitic lifestyle [75] and/or the presence of transposable elements [79,101] have been proposed in order to explain this dynamic arrangement.

In Rotifera, the genome survey of *Adineta vaga* demonstrated that *PG-3*, *Lox2* and posterior genes are absent while the other genes, with the exception of *PG-2*, present multiple copies. Moreover, not all the genes identified are arranged in a cluster [23]. The study in *Philodina roseola*, even if fragmentary, to date has identified only two central genes which present multiple copies (Table 1).

## 3. Expression Patterns

Insight into the presence of *Hox* genes and their expression patterns might be useful for better understanding the morphological innovations and the wide variety of lophotrochozoan species.

Most of the expression data on mollusks are limited to three species of gastropods [47,48,49,51,52], two species of cephalopods [46,56,102], one species of bivalves [24,38], and one species of chitons [57]. Data on the conchiferan species suggest the role of *Hox* genes in the central nervous system, their involvement in shell formation, tentacles, and the funnel; their expression has also been identified in sensory organs such as the apical organ and statocyst and in the light organ in the cephalopod *Euprymna scolopes*. Expression analysis in the bivalve *C. gigas* has shown that *Hox* genes are not activated according to temporal collinearity but that the peak in *PG-4*, *PG-1*, and *Lox4* expression occurs before gastrulation, while *Lox5* and *Post-2* are expressed during the trochophore stage, and the other genes in late development [24,38]. Recent findings in the aculiferan *Acanthochitona crinita* reveal a collinearity of *Hox* gene expression [57]. This is the first evidence in mollusks of the ancestral role of *Hox* genes in patterning the structures along the anterior-posterior body axis in the same way as most other bilaterian animals. Therefore, after the aculiferan-conchiferan split, *Hox* genes were co-opted into the formation of novelties in gastropods and cephalopods and perhaps in all the other conchiferan classes [57].

A comparison of expression patterns during trochophore larvae development in mollusks and annelids reveals that chitons share comparable expression patterns with annelids along the anterior-posterior body axis [57].

Expression surveys in the annelid Sedentaria confirm the ancestral role of *Hox* genes in determining the structures along the anterior-posterior body axis [8,62,67,70,73,103]. In particular, the study performed in *Capitella teleta* has shown that *Hox* genes exhibit spatial and temporal collinearity in line with the ancestral role that these genes had in the deuterostome-protostome ancestor [8]. However, the absence of collinearity observed in *H. robusta*, that shows a disrupted *Hox* cluster, indicates that collinearity is not conserved within Sedentaria [62]. The duplicated genes present in *P. excavatus* also seem to be expressed from the anterior to the posterior body region [67].

In the Errantia *N. virens* and *Platynereis dumerilii*, the expression patterns are very similar: all the *Hox* genes are involved in vectorial regionalization with the exception of *Post-1*; *PG-1*, *PG-4*, *PG-5*, *Lox5*, and *Post-2* are expressed in spatial collinearity while temporal collinearity is not respected since the Nereid body plan lacks unique segmental identities [70]. On the contrary, temporal collinearity has been detected in *Chaetopterus* *variopedatus* which occupies the basal position in Annelida [73].

The expression analysis of the nine *Hox* genes in the nemertean *M. alaskensis* clearly indicated that *Hox* genes are expressed not in the pilidial larva but in the juvenile stage of pilidiophorans [36]. The gene expression studies also performed on the hoplonemerteans *P. californiensis* revealed the homology between the imaginal discs of the pilidium and the paired larval invaginations in hoplonemerteans and showed that pilidial development evolved before the split between the two nemertean groups [37]. This sheds light on how *Hox* genes might be useful for understanding the evolution of embryonic development.

A high expression of *Hox* genes belonging to the paralog groups *PG-2*, *PG-4*, *Lox5*, *Lox4* and, to a lesser extent, *PG-1*, has been identified inside the eggs and at the miracidium stage in *Schistosoma* and in monogenean parasites [31,79,104], thus suggesting that these genes could be involved in embryo development. *Lox5* and *PG-4* genes have also exhibited a higher expression in the schistosomulum stage of *S. japonicum*, thereby highlighting the possible involvement of these two genes in multiple ontogenetic development stages in schistosomes [31,79]. Moreover, high expression of *PG-4*, *Lox5*, and *Lox4* has also been observed in sporocysts, another stage at which the determination of cell fate along the anterior-posterior axis may be important [79]. *Lox4* could be involved in the process of development in the monogenean parasite, given its high expression in the early developmental stages of the branchial phenotype [104]. During embryogenesis and embryo patterning in the planarian *Schmidtea polychroa,* the *Spol-hoxD* (*Lox5*) gene transcripts started to be detected at early stage 6 (8–10 days), in a strip of cells on the side of the embryo containing the definitive pharynx; the pattern spreads from the definitive pharynx to the posterior end, as reported by Iglesias *et al.* [89] in the adult. These data suggest that the adult anterior-posterior axis is established after yolk ingestion and the proliferation of the blastomeres in the germ band (stage 5) [90].

Studies on *Hox* expression have also been performed in adults. The patterns in adult specimens of *Girardia tigrina,* *Discocelis tigrina,* and *Dugesia japonica* have shown two types of *Hox* genes, that either conserve or lose their typical differential spatial expression whereas duplicated genes may show both patterns of expression [84,105]. In the planarian *D. japonica,* Nogi and Watanabe [83] reported a similar expression pattern for the two *Post-2* genes: *DjAbd-Ba* is expressed from the posterior pharyngeal region to the entire tail region suggesting that this gene is involved in the specification of the tail region, while *DjAbd-Bb* is expressed in several types of cells throughout the body. However, counter to the rule of spatial collinearity, the anterior boundary of the expression domain of the posterior gene *DjAbd-Ba* is anterior to the domains of the central genes *PG-4* and *Lox5*. Different functions have also been reported for *Hox* genes, apart from their involvement in embryonic development; for example, it has been hypothesized that *Lox4*, being highly expressed in adult males and down-regulated in adult females, could be involved in tissue differentiation of the male reproductive tract [104]. Another feature is the permanent *Hox* expression in adult organisms; the high morphological plasticity of these organisms, related to the presence in adult organisms of neoblasts (undifferentiated and totipotent cell types, [106]), could explain their activity, suggesting that pattern formation in planarians may occur continuously [82,105].

As well as embryonic development, the anterior-posterior positional values of *Hox* genes are also involved in regeneration. After wounding, regenerative tissue is quickly formed [106], accompanied by cell proliferation and *Hox* expression, even if the two processes are not necessarily related [86]. It has been pointed out that during regeneration, only *Lox5* and *Post-2* genes have a differential axial nested expression, while the other genes are ubiquitously expressed. These genes are activated during the first day of tail regeneration and down-regulated during head regeneration [82,83,105,107,108]. In particular, the presence of a system that maintains anterior-posterior axial polarity and regulates the expression of *DjAbd-Ba* rapidly after amputation could be confirmed by the rapid expression of *DjAbd-Ba* (*Post-2a*) in the head piece after amputation, with the anterior boundary of the *DjAbd-Ba* expression domain shifting rapidly and dynamically toward the posterior in the tail pieces [83]. Moreover, *Hox* gene expression has also been detected during lateral regeneration underlining the importance of these genes in regenerative processes in order to specify positional information on any axis [86].

The extra copies of *Hox* genes identified in some Platyhelminthes may have lost their typical anterior-posterior axial patterning role as a result of independent duplication and may, on the contrary, have acquired a function in cell differentiation [79,82,86].

In general, flatworm *Hox* genes have been studied for many years, and, in some cases, distinct spatial domains of expression have been highlighted, although their specific functions have not yet been identified. Further research studies, especially on embryo development, are required in order to draw some conclusions on the role and the possible expression collinearity of *Hox* genes in Platyhelminthes.

## 4. Conclusions

This review provides clear evidence that insight into *Hox* cluster composition and expression patterns is limited to a few phyla and that information is completely lacking for about half the taxa belonging to Lophotrochozoa.

The overview of the literature considered in this work indicates that *Lox2* and *Post-1* genes have only been recovered in some taxa of Lophotrochozoa. In addition to the hypothesis of a common ancestor with 11 genes, a further suggestion is that these two genes originated as a result of secondary duplications and that the common ancestor had nine genes [36,37,94].

If the latter hypothesis is valid the duplications of the central *Lox4* and *Lox2* genes and the posterior *Post-1* and *Post-2* genes must have occurred in the ancestor of Mollusca-Annelida-Brachiopoda. As regards Nemerteans, if their position is within this clade, the lack of *Lox2* and *Post-1* genes (since they have not been identified to date) may be due to a secondary loss. Moreover, in Platyhelminthes, *PG-5* and *Antp* have also been lost, thereby suggesting that additional gene losses have occurred compared with the common ancestor.

In Mollusca and Annelida, a complete spatial collinearity involving all the genes that make up the *Hox* cluster has only been detected in the polyplacophoran *Acanthochitona* and in the annelid *Capitella*, while in other species of annelids, such as *Helobdella triserialis, Nereis virens*, and *Platynereis dumerilii,* only some *Hox* genes maintain spatial collinearity [62,70].

This suggests that, during evolution, the collinearity of *Hox* gene expression has been maintained only in some taxa of these phyla. In flatworms, in which the breakage of the cluster and the loss of some genes occurred, spatial-temporal collinearity seems to have been lost. Since *Hox* genes are expressed in spatial collinearity in lophotrochozoans, ecdysozoans, and deuterostames, the most parsimonious conclusion is that the spatial collinearity of *Hox* genes was already a feature of the last common bilaterian ancestor.

Duplicated genes have also been identified in Clitellata and in Platyhelminthes, and the extra copies of *Hox* genes show different expression patterns suggesting neofunctionalizations.

The comparison of *Hox* cluster composition and expression patterns in various animal groups is a pivotal step toward understanding the mechanisms by which body plan modifications occurred determining animal radiation. Therefore, future studies should be focused on the identification and expression of *Hox* genes in phyla and classes which either have not yet been analyzed or which have received scarce attention (Micrognathozoa, Acanthocephala, Rotifera, Entoprocta, Cycliophora, and Phoronida). Furthermore, the information about some taxa is restricted to a single species and, therefore, more species need to be investigated so as to have a better overview.

## Figures and Tables

**Table 1:** *Hox* genes identified to date in Brachiopoda, Bryozoa, Rotifera, and Nemertea.

		*Hox* Genes											
Phylum	Species	*PG-1*	*PG-2*	*PG-3*	*PG-4*	*PG-5*	*Lox5*	*Antp*	*Lox2*	*Lox4*	*Post-1*	*Post-2*	References
**Brachiopoda**	*Lingula anatina*	√	√	√	√	√	√	√	√	√	√	√	[16,22]
**Bryozoa**	*Bugula turrita*		√	√	√ *		√					√	[34]
**Rotifera**	*Adineta vaga*	√ *	√		√ *	√ *	√ *	√ *		√ *			[23]
	*Philodina roseola*					√ *	√ *						[91]
**Nemertea**	*Lineus sanguineus*	√		√	√		√	√				√	[35]
	*Micrura alaskensis*	√	√	√	√	√	√	√		√		√	[36]
	*Pantinonemertes californiensis*	√	√	√	√		√					√	[37]

* indicates duplicated genes.

## Supplementary Materials

Supplementary are available online at www.mdpi.com/2221-3759/4/1/12/s1.
