# Supplementary material for: An Overview of Hox Genes in Lophotrochozoa: Evolution and Functionality
Source: J Dev Biol. 2016 Mar 19;4(1):12. doi: 10.3390/jdb4010012 (PMC5831810; doi:10.3390/jdb4010012)
Supplement: Supplementary file 1 [file jdb-04-00012-s001.pdf]

Supplementary Materials: An Overview of *Hox* Genes in Lophotrochozoa: Evolution and Functionality

Marco Barucca, Adriana Canapa and Maria Assunta Biscotti

Table S1. *Hox* genes identified to date in Mollusca.

						**Mollusca**							
						***Hox* Genes**							
**Class**	**Species**	***PG-1***	***PG-2***	***PG-3***	***PG-4***	***PG-5***	***Lox5***	***Antp***	***Lox2***	***Lox4***	***Post-1***	***Post-2***	**References**
**Bivalvia**	*Crassostrea gigas*	√	√	√	√	√	√		√	√	√	√	[1,2]
	Ensis ensis			√		√							[3]
	Mimachlamys varia						√				√	√	[4]
	Mizuhopecten yessoensis	√	√	√	√	√ *		√	√	√	√	√	[5]
	Mytilus edulis	√			√	√							[6]
	Mytilus galloprovincialis	√	√	√	√								[3,7]
	Ostrea edulis	√				√						√	[6]
	Pecten maximus	√	√	√	√	√	√	√	√	√	√	√	[3,8,9]
	Pinctada fucata	√	√	√	√	√	√	√	√	√	√	√	[10,11]
	Solen marginatus	√	√	√		√	√			√			[12]
	Tapes philippinarum						√			√			[3]
	Yoldia eightsi		√					√					[3]
	Venerupis pullastra	√		√		√	√	√					[12]
**Gastropoda**	*Aplysia californica*					√		√			√		[13]
	Bulla bulla		√										[14]
	Gibbula varia	√	√	√	√	√	√	√	√	√	√	√	[15,16]
	Haliotis asinina	√	√	√	√	√							[17–19]
	Haliotis rufescens	√	√		√	√							[17,20]
	Kelletia kelletii	√											[14]
	Ilyanassa obsoleta		√				√						[21]
	Lottia gigantea	√	√	√	√	√	√	√	√	√	√	√	[22]

	Lymnaea stagnalis	√	√	√		√		√ *	√	√	√	√	[5]
	Patella vulgata			√		√	√	√	√	√	√		[23,24]
**Scaphopoda**	*Dentalium octangulatum*	√	√	√	√	√ *			√	√	√	√	[5]
**Cephalopoda**	*Euprymna scolopes*	√		√		√	√	√		√	√	√	[14,25]
	Nautilus macromphalus				√	√	√	√	√				[26]
	Nautilus pompilius	√	√	√		√ *		√ *	√	√	√	√	[5,26]
	Octopus bimaculoides	√				√	√	√	√	√	√	√	[27]
	Sepia officinalis	√		√		√				√		√	[26]
**Polyplacophora**	*Acanthochitona crinita*	√	√	√	√	√		√				√	[28]
	Acanthopleura japonica	√				√ *		√	√			√	[5]
	Nuttallochiton mirandus	√	√			√	√	√	√		√	√	[29]
**Caudofoveata**	*Chaetoderma japonica*			√		√ *			√	√			[5]
**Solenogastres**	*Unidentified* sp.	√	√	√		√ *	√		√		√	√	[5]

* indicates duplicated genes probably due to erroneous attribution [30].

Table S2. *Hox* genes identified to date in Annelida.

Annelida												
Hox Genes												
**Species**	***PG-1***	***PG-2***	***PG-3***	***PG-4***	***PG-5***	***Lox5***	***Antp***	***Lox2***	***Lox4***	***Post-1***	***Post-2***	**References**
Capitella teleta	√	√	√	√	√	√	√	√	√	√	√	[22,31]
Ctenodrilus serratus	√	√	√	√	√	√	√	√	√			[32]
Eisenia andrei	√		√	√	√	√	√	√	√	√	√	[33]
Eisenia fetida	√ *		√ *	√	√ *	√	√	√ *	√ *	√ *	√ *	[34]
Helobdella robusta	√ *		√	√ *	√ *	√	√	√	√ *		√ *	[22]
Helobdella triserialis	√			√	√							[35]
Hirudo medicinalis				√	√			√	√			[36–39]
Lombricus sp.			√									[14]

	Perionyx excavatus	√ *	√	√ *	√ *	√	√	√	√	√	√	√ *	[40]
	Pristina longiseta	√ *		√									[41]
	Stylaria lacustris	√ *		√									[42]
	Tubifex tubifex	√		√	√	√ *	√		√ *	√			[43]
Errantia	*Nereis virens*	√	√	√	√	√	√	√	√	√	√	√	[23,44]
	Platynereis dumerilii	√	√	√	√	√	√		√		√	√	[45]
	Myzostoma cirriferum	√	√		√		√	√		√		√	[46]
	Urechis caupo			√									[14]
	Urechis unicinetus	√ *		√	√	√		√	√	√		√	[47]
	Chaetopterus variopedatus	√		√	√	√	√	√	√	√			[48]

* indicates duplicated genes.

Table S3. *Hox* genes identified to date in Platyhelminthes.

Platyhelminthes													
***Hox* Genes**													
**Class/Order**	**Species**	*PG-1*	*PG-2*	*PG-3*	*PG-4*	*PG-5*	*Lox5*	*Antp*	*Lox2*	*Lox4*	*Post-1*	*Post-2*	**References**
**Cestoda**	*Echinococcus granulosus*	√		√	√		√					√	[49]
	Echinococcus multilocularis	√		√	√		√			√		√ *	[50,51]
	Echinococcus vogeli	√											[52]
	Hymenolepis microstoma	√			√		√			√		√ *	[51,53]
	Mesocestoides corti	√								√			[50]
	Mesocestoides vogae	√					√			√			[54]
	Taenia asiatica	√ *		√ *	√					√ *			[55]
	Taenia solium	√		√	√		√			√		√ *	[51]
**Trematoda**	*Echinostoma trivolvis*	√		√	√					√			[53,56]
	Fasciola hepatica	√			√								[52]
	Schistosoma haematobium	√			√								[57]
	Schistosoma japonicum	√	√	√	√		√			√		√ *	[58]
	Schistosoma mansoni	√	√	√	√		√ *			√		√ *	[50,59,60]
**Monogenea**	*Concinnocotyla australensis*	√		√						√			[61]
	Diclidophora luscae			√	√					√			[61]
	Gyrodactylus salaris	√	√ *		√		√			√		√ *	[62]
	Polystoma gallieni									√ *			[61]
	Polystomoides malayi			√ *									[61]
	Polystomoides coronatum	√		√	√								[61]
	Protopolystoma xenopodis	√			√								[61]

	Pseudodiplorchis americanus					√	√		[61]
	Pseudopolystoma dendriticum	√		√	√		√ *		[61]
	Sphyranura oligorchis			√			√		[61]
**Tricladida**	*Dendrocoelum lacteum*	√		√			√		[63]
	Dugesia japonica			√	√	√ *		√ *	[50,64–66]
	Dugesia ryukyuensis							√	[67]
	Girardia tigrina		√	√	√	√ *	√		[50,66,68–70]
	Phagocata woodworthi	√ *		√			√ *		[56]
	Polycelis felina	√ *					√		[63]
	Polycelis nigra	√ *		√ *	√	√	√ *		[63,71]
	Schmidtea mediterranea					√		√	[72]
	Schmidtea polychroa					√			[73]
**Polycladida**	*Discocelis tigrina*	√			√		√		[66]
**Macrostomida**	*Macrostoma lignano*	√ *		√		√		√	[74]

* indicates duplicated genes. The duplication reported for *PG-2* gene in *Gyrodactylus salaris* might be due to an erroneous attribution of *PG*.

References

1. Zhang, G.; Fang, X.; Guo, X.; Li, L.; Luo, R.; Xu, F.; Yang, P.; Zhang, L.; Wang, X.; Qi, H.; *et al*. The oyster genome reveals stress adaptation and complexity of shell formation. *Nature* **2012**, *490*, 49–54.
2. Paps, J.; Xu, F.; Zhang, G.; Holland, P.W. Reinforcing the egg-timer: Recruitment of novel lophotrochozoa homeobox genes to early and late development in the pacific oyster. *Genome Biol. Evol.* **2015**, *7*, 677–688.
3. Barucca, M.; Olmo, E.; Canapa, A. *Hox* and *ParaHox* genes in bivalve molluscs. *Gene* **2003**, *317*, 97–102.
4. Pérez-Parallé, L.; Mesias-Gansbiller, C.; Sanchez, J.L. University of Santiago de Compostela, Santiago de Compostela, Spain. Unpublished work, 2009.
5. Iijima, M.; Akiba, N.; Sarashina, I.; Kuratani, S.; Endo, K. Evolution of *Hox* genes in molluscs: A comparison among seven morphologically diverse classes. *J. Molluscan Stud.* **2006**, *72*, 259–266.
6. Mesías-Gansbiller, C.; Sánchez, J.L.; Pazos, A.J.; Lozano, V.; Martínez-Escauriaza, R.; Luz Pérez-Parallé, M. Conservation of *Gbx* genes from EHG homeobox in bivalve molluscs. *Mol. Phylogenet. Evol.* **2012**, *63*, 213–217.
7. Peréz-Parallé, M.L.; Carpintero, P.; Pazos, A.J.; Abad, M.; Sánchez, J.L. The *Hox* cluster in the bivalve mollusc *Mytilus galloprovincialis*. *Biochem. Genet.* **2005**, *43*, 417–424.
8. Canapa, A.; Biscotti, M.A.; Olmo, E.; Barucca, M. Isolation of *Hox* and *ParaHox* genes in the bivalve *Pecten maximus*. *Gene* **2005**, *348*, 83–88.
9. Carpintero, P.; Pazos, A.J.; Abad, M.; Sánchez, J.L.; Pérez-Parallé, M.L. Presence *Proboscipedia* and *Caudal* gene homologues in a bivalve mollusc. *J. Biochem. Mol. Biol.* **2004**, *37*, 625–628.
10. Takeuchi, T.; Koyanagi, R.; Gyoja, F.; Kanda, M.; Hisata, K.; Fujie, M.; Goto, H.; Yamasaki, S.; Nagai, K.; Morino, Y.; *et al*. Bivalve-specific gene expansion in the pearl oyster genome: Implications of adaptation to a sessile lifestyle. *Zool. Lett*. **2016**, *2*, doi:10.1186/s40851-016-0039-2.
11. Morino, Y.; Okada, K.; Niikura, M.; Honda, M.; Satoh, N.; Wada H. A genome-wide survey of genes encoding transcription factors in the Japanese pearl oyster, *Pinctada fucata*: I. Homeobox genes. *Zool. Sci.* **2013**, *30*, 851–857.
12. Pérez-Parallé, L.; Mesias-Gansbiller, C.; Sanchez, J.L. University of Santiago de Compostela, Santiago de Compostela, Spain. Unpublished work, 2011.
13. BioprojectPRJNA209509, Broad Institute, Cambridge, MA, USA. Unpublished work, 2006.
14. Lee, S.E.; Gates, R.D.; Jacobs, D.K. Gene fishing: The use of a simple protocol to isolate multiple homeodomain classes from diverse invertebrate taxa. *J. Mol. Evol.* **2003**, *56*, 509–516.
15. Samadi, L.; Steiner, G. Involvement of *Hox* genes in shell morphogenesis in the encapsulated development of a top shell gastropod (*Gibbula varia* L.). *Dev. Genes Evol.* **2009**, *219*, 523–530.
16. Samadi, L.; Steiner, G. Expression of *Hox* genes during the larval development of the snail, *Gibbula varia* [L]-further evidence of non-colinearity in molluscs. *Dev. Genes Evol.* **2010**, *220*, 161–172.
17. Giusti, A.F.; Hinman, V.F.; Degnam, S.M.; Degnan, B.M.; Morse, D.E. Expression of a *Scr/Hox5* gene in the larval central nervous system of the gastropod *Haliotis*, a non-segmented spiralian lophotrochozoan. *Evol. Dev.* **2000**, *2*, 294–302.
18. Hinman, V.F.; Degnan, B.M. *Mox* homeobox expression in muscle lineage of the gastropod *Haliotis asinina*: Evidence for a conserved role in bilaterian myogenesis. *Dev. Genes Evol.* **2002**, *212*, 141–144.
19. Hinman, V.F.; O'Brien, E.K.; Richards, G.S.; Degnan, B.M. Expression of anterior *Hox* genes during larval development of the gastropod *Haliotis asinina*. *Evol. Dev.* **2003**, *5*, 508–521.
20. Degnan, B.M.; Morse, D.E. Identification of eight homeobox-containing transcripts expressed during larval development and at metamorphosis in the gastropod mollusc *Haliotis rufescens*. *Mol. Mar. Biol. Biotechnol.* **1993**, *2*, 1–9.
21. Lambert, J.D.; Chan, X.Y.; Spiecker, B.; Sweet, H.C. Characterizing the embryonic transcriptome of the snail *Ilyanassa*. *Integr. Comp. Biol.* **2010**, *50*, 768–777.
22. Simakov, O.; Marletaz, F.; Cho, S.-J.; Edsinger-Gonzales, E.; Havlak, P.; Hellsten, U.; Kuo, D.H.; Larsson, T.; Lv, J.; Arendt, D.; *et al*. Insights into bilaterian evolution from three spiralian genomes. *Nature* **2013**, *493*, 526–531.
23. De Rosa, R.; Grenier, J.K.; Andreeva, T.; Cook, C.E.; Adoutte, A.; Akam, M.; Carroll, S.B.; Balavoine, G. *Hox* genes in brachiopods and priapulids and protostome evolution. *Nature* **1999**, *399*, 772–776.
24. Kenny, N.J.; Namigai, E.K.; Marlétaz, F.; Hui, J.H.; Shimeld, S.M. Draft genome assemblies and predicted microRNA complements of the intertidal lophotrochozoans *Patella vulgata* (Mollusca, Patellogastropoda) and *Spirobranchus* (*Pomatoceros*) *lamarcki* (Annelida, Serpulida). *Mar. Genom.* **2015**, *24 Pt 2*, 139–146.

25. Callaerts, P.; Lee, P.N.; Hartmann, B.; Farfan, C.; Choy, D.W.; Ikeo, K.; Fischbach, K.-F.; Gehring, W.J.; Gert de Couet, H. *Hox* genes in the sepiolid squid *Euprymna scolopes*: Implications for the evolution of complex body plans. *Proc. Natl. Acad. Sci. USA* **2002**, *99*, 2088–2093.
26. Pernice, M.; Deutsch, J.S.; Andouche, A.; Boucher-Rodoni, R.; Bonnaud, L. Unexpected variation of *Hox* genes' homeodomains in cephalopods. *Mol. Phylogenet. Evol.* **2006**, *40*, 872–879.
27. Albertin, C.B.; Simakov, O.; Mitros, T.; Wang, Z.Y.; Pungor, J.R.; Edsinger-Gonzales, E.; Brenner, S.; Ragsdale, C.W.; Rokhsar, D.S. The octopus genome and the evolution of cephalopod neural and morphological novelties. *Nature* **2015**, *524*, 220–224.
28. Fritsch, M.; Wollesen, T.; de Oliveira, A.L.; Wanninger, A. Unexpected co-linearity of *Hox* gene expression in an aculiferan mollusk. *BMC Evol. Biol.* **2015**, *15*, doi:10.1186/s12862-015-0414-1.
29. Biscotti, M.A.; Canapa, A.; Olmo, E.; Barucca, M. *Hox* genes in the Antarctic polyplacophoran *Nuttallochiton mirandus*. *J. Exp. Zool. Part B* **2007**, *308*, 507–513.
30. Biscotti, M.A.; Canapa, A.; Forconi, M.; Barucca, M. *Hox* and *ParaHox* genes: A review on molluscs. *Genesis* **2014**, *52*, 935–945.
31. Fröbius, A.C.; Matus, D.Q.; Seaver, E.C. Genomic organization and expression demonstrate spatial and temporal *Hox* gene colinearity in the lophotrochozoan *Capitella sp I*. *PLoS ONE* **2008**, *3*, e4004.
32. Dick, M.H.; Buss, L.W. A PCR-based Survey of Homeobox Genes in *Ctenodrilus serratus* (Annelida: Polychaeta). *Mol. Phylogenet. Evol.* **1994**, *3*,146–158.
33. Cho, P.Y.; Cho, S.J.; Lee, M.S.; Lee, J.A.; Tak, E.S.; Shin, C.; Choo, J.K.; Park, S.C.; Lee, K.-S.; Park, H-Y.; *et al*. Note: A PCR-Based Analysis of *Hox* Genes in an Earthworm, *Eisenia andrei* (Annelida: Oligochaeta). *Biochem. Genet.* **2004**, *42*, 209–216.
34. Zwarycz, A.S.; Nossa, C.W.; Putnam, N.H.; Ryan, J. Timing and scope of genomic expansion within Annelida: Evidence from homeoboxes in the genome of the earthworm *Eisenia fetida*. *Genome Biol. Evol.* **2015**, doi: 10.1093/gbe/evv243.
35. Kourakis, M.J.; Master, V.A.; Lokhorst, D.K.; Nardelli-Haefliger, D.; Wedeen, C.J.; Martindale, M.Q.; Shankland, M. Conserved anterior boundaries of *Hox* gene expression in the central nervous system of the leech *Helobdella*. *Dev. Biol.* **1997**, *190*, 284–300.
36. Aisemberg, G.O.; Macagno, E.R. *Lox1*, an Antennapedia-class homeobox gene, is expressed during leech gangliogenesis in both transient and stable central neurons. *Dev. Biol.* **1994**, *161*, 455–465.
37. Wong, V.Y.; Aisemberg, G.O.; Gan, W.B.; Macagno, E.R. The leech homeobox gene *Lox4* may determine segmental differentiation of identified neurons. *J. Neurosci.* **1995**, *15*, 5551–5559.
38. Wong, V.Y.; Macagno, E.R. *Lox6*, a leech *Dfd* ortholog, is expressed in the central nervous system and in peripheral sensory structures. *Dev. Genes Evol.* **1998**, *208*, 51–55.
39. Wysocka-Diller, J.W.; Aisemberg, G.O.; Baumgarten, M.; Levine, M.; Macagno, E.R. Characterization of a homologue of bithorax-complex genes in the leech *Hirudo medicinalis*. *Nature* **1989**, *341*, 760–763.
40. Cho, S.J.; Vallès, Y.; Kim, K.M.; Ji, S.C.; Han, S.J.; Park, S.C. Additional duplicated *Hox* genes in the earthworm: *Perionyx excavatus Hox* genes consist of eleven paralog groups. *Gene* **2012**, *493*, 260–266.
41. Kostyuchenko, R.P. State University of St. Petersburg, St. Petersburg, Russia. Unpublished work, 2013.
42. Snow, P.; Buss, L.W. HOM/*Hox*-type homeoboxes from *Stylaria lacustris* (Annelida: Oligochaeta). *Mol. Phylogenet. Evol.* **1994**, *3*, 360–364.
43. Shimizu, T.; Sakai, C.; Endo, M. Takashi Shimizu Hokkaido University, Sapporo, Japan. Unpublished work, 2013.
44. Andreeva, T.F.; Cook, C.; Korchagina, N.M.; Akam, M.; Dondua, A.K. Cloning and analysis of structural organization of *Hox* genes in the polychaete *Nereis virens*. *Ontogenez* **2001**, *32*, 225–233.
45. Kulakova, M.; Bakalenko, N.; Novikova, E.; Cook, C.E.; Eliseeva, E.; Steinmetz, P.R.; Kostyuchenko, R.P.; Dondua, A.; Arendt, D.; Akam, M.; *et al. Hox* gene expression in larval development of the polychaetes *Nereis virens* and *Platynereis dumerilii* (Annelida, Lophotrochozoa). *Dev. Genes Evol.* **2007**, *217*, 39–54.
46. Bleidorn, C.; Lanterbecq, D.; Eeckhaut, I.; Tiedemann, R. A PCR survey of *Hox* genes in the myzostomid *Myzostoma cirriferum*. *Dev. Genes Evol.* **2009**, *219*, 211–216.
47. Cho, S.J.; Lee, D.H.; Kwon, H.J.; Ahn, C.H.; Park, S.C.; Shin, K.S. *Hox* genes in the echiuroid *Urechis unicinctus*. *Dev. Genes Evol.* **2006**, *216*, 347–351.
48. Irvine, S.Q.; Martindale, M.Q. Expression patterns of anterior *Hox* genes in the polychaete *Chaetopterus*: Correlation with morphological boundaries. *Dev. Biol.* **2000**, *217*, 333–351.
49. Zheng, H.; Zhang, W.; Zhang, L.; Zhang, Z.; Li, J.; Lu, G.; Zhu, Y.; Wang, Y.; Huang, Y.; Liu, J.; *et al*. The genome of the hydatid tapeworm *Echinococcus granulosus*. *Nat. Genet*. **2013**, *45*, 1168–1175.

50. Koziol, U.; Lalanne, A.I.; Castillo, E. *Hox* genes in the parasitic Platyhelminthes *Mesocestoides corti*, *Echinococcus multilocularis*, and *Schistosoma mansoni*: Evidence for a reduced *Hox* complement. *Biochem. Genet*. **2009**, *47*, 100–116.
51. Tsai, I.J.; Zarowiecki, M.; Holroyd, N.; Garciarrubio, A.; Sánchez-Flores, A., Brooks, K.L.; Tracey, A.; Bobes, R.J.; Fragoso, G.; Sciutto, E.; *et al*. The genomes of four tapeworm species reveal adaptations to parasitism. *Nature* **2013**, *496*, 57–63.
52. Oliver, G., Vispo, M.; Mailhos, A.; Martinez, C.; Sosa-Pineda, B.; Fielitz, W.; Ehrlich, R. Homeoboxes in flatworms. *Gene* **1992**, *121*, 337–342.
53. Olson, P.D. Hox genes and the parasitic flatworms: New opportunities, challenges and lessons from the free-living. *Parasitol. Int*. **2008**, *57*, 8–17.
54. Castillo, E.; Lalanne, A.I.; Chalar, C.; Martinez, C.; Ehrlich, R. Faculty of Sciences, Montevideo, Uruguay. Unpublished work, 2003.
55. Kim, K.H.; Lee, Y.S.; Jeon, H.K.; Park, J.K.; Kim, C.B.; Eom, K.S. *Hox* genes from the tapeworm *Taenia asiatica* (Platyhelminthes: Cestoda). *Biochem. Genet*. **2007**, *45*, 335–343.
56. Bartels, J.L.; Murtha, M.T.; Ruddle, F.H. Multiple Hox/HOM-class homeoboxes in Platyhelminthes. *Mol. Phylogenet. Evol*. **1993**, *2*, 143–151.
57. Young, N.D.; Jex, A.R.; Li, B.; Liu, S.; Yang, L.; Xiong, Z.; Li, Y.; Cantacessi, C.; Hall, R.S.; Xu, X.; *et al*. Whole-genome sequence of *Schistosoma haematobium. Nat. Genet*. **2012**, *44*, 221–225.
58. Gu, J.L.; Chen, S.X.; Dou, T.H.; Xu, M.J.; Xu, J.X.; Zhang, L.; Hu, W.; Wang, S.J.; Zhou, Y. *Hox* genes from the parasitic flatworm *Schistosoma japonicum*. *Genomics* **2012**, *99*, 59–65.
59. Webster, P.J.; Mansour, T.E. Conserved classes of homeodomains in Schistosoma mansoni, an early bilateral metazoan. *Mech. Dev*. **1992**, *38*, 25–32.
60. Pierce, R.; Wu, W.; Hirai, H.; Ivens, A.; Murphy, L.D.; Noël, C.; Johnston, D.A.; Artiguenave, F.; Adams, M.; Cornette, J.; *et al*. Evidence for a dispersed *Hox* gene cluster in the platyhelminth parasite *Schistosoma mansoni*. *Mol. Biol. Evol*. **2005**, *22*, 2491–503.
61. Badets, M.; Verneau, O. *Hox* genes from Polystomatidae (Platyhelmintes, Monogenea). *Int. J. Parassitol.* **2009**, *39*, 1517–1523.
62. Hahn, C.; Fromm, B.; Bachmann, L. Comparative genomics of flatworms (Platyhelminthes) reveals shared genomic features of ecto- and endoparastic Neodermata. *Genome Biol. Evol.* **2015**, *6*, 1105–1117.
63. Balavoine, G.; Telford, M.J. Identification of planarian homeobox sequences indicates the antiquity of most *Hox*/homeotic gene subclasses. *Proc. Natl. Acad. Sci. USA.* **1995**, *92*, 7227–7231.
64. Orii, H.; Kato, K.; Umesono, Y.; Sakurai, T.; Agata, K.; Watanabe, K. The planarian HOM/*HOX* homeobox genes (*Plox*) expressed along the anteroposterior axis. *Dev. Biol.* **1999**, *210*, 456–468.
65. Nogi, T.; Watanabe, K. Position-specific and non-colinear expression of the planarian posterior (Abdominal-B-like) gene. *Dev. Growth Differ.* **2001**, *43*, 177–184.
66. Saló, E.; Tauler, J.; Jmenéz, E.; Bayascas, J.R.; González-Linares, J.; Garcia-Fernàndez, J.; Baguñà, J. *Hox* and *ParaHox* Genes in Flatworms. Characterization and Expression. *Am. Zool.* **2001**, *41*, 652–663.
67. Matsumoto, M.; Nakagawa, H. Keio University, Yokohama, Japan. Unpublished work, 2005.
68. Tarabykin, V.S.; Lukyanov, K.A.; Potapov, V.K.; Lukyanov, S.A. Detection of planarian Antennapedialike, homeobox genes expressed during regeneration. *Gene* **1995**, *158*, 197–202.
69. Bayascas, J.R.; Castillo, E.; Muñoz-Mármol, A.M.; Saló, E. Planarian *Hox* genes: Novel patterns of expression during re generation. *Development* **1997**, *124*, 141–148.
70. Bayascas, J.R.; Castillo, E.; Saló, A.M. Platyhelminthes have a *Hox* code differentially activated during regeneration, with genes closely related to those of spiralian protostomes. *Dev. Genes Evol.* **1998**, *208*, 467–473.
71. Balavoine, G. Identification of members of several homeobox genes in a planarian using a ligation-mediated polymerase chain reaction technique. *Nucleic Acids. Res.* **1996**, *8*, 1547–1553.
72. Iglesias, M.; Gomez-Skarmeta, J.L.; Saló, E.; Adell, T. Silencing of *Smed-catenin1* generates radial-like hypercephalized planarians. *Development* **2008**, *135*, 1215–1221.
73. Martín-Durán, J.M.; Amaya, E.; Romero, R. Germ layer specification and axial patterning in the embryonic development of the freshwater planarian *Schmidtea polychroa. Dev. Biol.* **2010**, *340*, 145–158.
74. Wasik, K.; Gurtowski, J.; Zhou, X.; Ramos, O.M.; Delás, M.J.; Battistoni, G.; El Demerdash, O.; Falciatori, I.; Vizoso, D.B.; Smith, A.D.; *et al*. Genome and transcriptome of the regeneration-competent flatworm, *Macrostomum lignano*. *Proc. Natl. Acad. Sci. USA* **2015**, *112*, 12462–12467.
